# A Novel High-Throughput Assay Reveals That the Temperature Induced Increases in Transphosphatidylation of Phospholipase D Are Dependent on the Alcohol Acceptor Concentration

**DOI:** 10.3390/biom12050632

**Published:** 2022-04-25

**Authors:** Hengzhang Yang, Rüdiger Woscholski

**Affiliations:** Department of Chemistry, Molecular Sciences Research Hub, White City Campus, Imperial College London, London W12 0BZ, UK; h.yang15@imperial.ac.uk

**Keywords:** phospholipase D, enzyme-assisted synthesis, allosteric, temperature, alcohol, lipid

## Abstract

Phospholipase D reacts with alcohols or water, transphosphatidylating or hydrolysing lipids such as phosphatidylcholine, generating phosphatidylalcohols or phosphatidic acid, respectively. The enzyme has been employed in many applications making use of the transphosphatidylation reaction and the enzyme’s tolerance for organic solvents in order to synthesize natural and artificial phospholipids. Yet, its catalytic properties with respect to the transphosphatidylation reaction are not well understood. Here, we introduce a novel high-throughput assay, making use of 96-well plates, that employs Fluorescamine for the detection of transphosphatidylated amino alcohols. This assay allowed to monitor the *K*_M_ and *V*_Max_ at different temperatures, revealing that the former will be elevated by the temperature, while the latter is increased by a combination of both temperature and alcohol acceptor concentration being elevated, suggesting that increase in temperature may open up a new binding site for the alcohol acceptor.

## 1. Introduction

**Figure 1:**
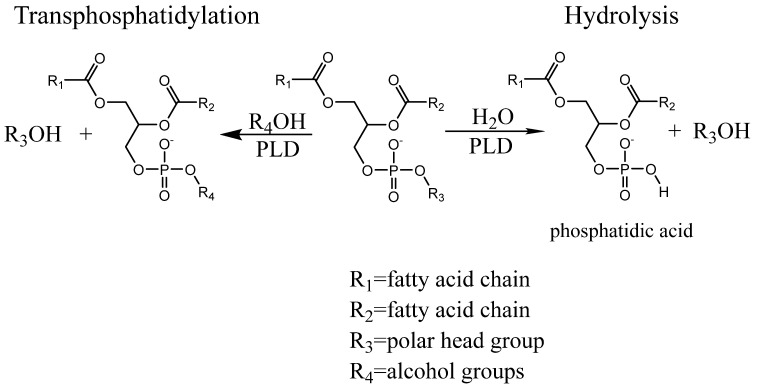
Hydrolysis and transphosphatidylation catalysed by PLD.

Biocatalysts are important alternatives to chemical catalysts providing environmental compatibilities that the latter lack. They are significant components towards adapting a greener chemistry and supporting sustainable development and circular economies in the future [1]. While there is a wide range of research currently looking into employing enzymes that can be exploited to catalyse reactions beyond their “natural” scope, their main focus seems to be on a hydrolytic reactions, trans-aminations, redox reactions, and halogenations [1,2], which have been increasingly adapted by the biotech and pharmaceutical industries [3]. Currently, a variety of accessible enzymes including phospholipase D (PLD) are utilised for chemical reactions. PLDs are phosphodiesterases catalysing the removal of the phospholipid headgroups through hydrolysis but can also, in the presence of alcohols, exchange the lipid headgroup with the provided alcohol (Figure 1). Enzymes catalysing this particular reaction are either belonging to the PLD superfamily based on a conserved HKD motif in the catalytic site, or a part of the non-HKD PLDs, which include Glycosylphosphatidylinositol-PLDs, N-acylphosphatidylethanolamine PLDs as well as autotaxins. PLDs in eukaryotic cells are involved in intra-cellular signalling, while bacterial PLDs being often secreted and essential for the virulence of these organisms [4]. These secreted bacterial PLDs show stronger transphosphatidylation potential than their eukaryotic siblings, making them ideal for the catalysis of transphosphatylation in the biotech sector [5,6,7]. In particular, an HKD-PLD derived from the organism *Streptomyces* sp. showed a high preference for the transphosphatidylation reaction (compared to the hydrolysis reaction) with a variety of different alcohol acceptors. This includes primary alcohols, as is the case for the majority of other PLDs, but also secondary alcohols [8], including nucleotides or sugars [9] as well as aromatic alcohols albeit with much reduced yield [10]. The artificial phospholipids created by the transphosphatidylation reaction catalysed by PLD from *Streptomyces* sp. could be potential drugs favouring specific uptake routes such as the lymph system [11] or building blocks for synthetic biology investigations [12].

Most of the studies so far have been concentrating on a few bacterial PLDs with the PLD derived from *Streptomyces* sp. being widely employed due to its tolerance for organic solvents and high transphosphatidylation activity [7]. However, not much attention has been on the principles governing the catalytic efficiencies of this PLD, focussing instead on the yield of the enzyme-assisted synthesis. Thus, the current understanding is mainly driven by experimental data obtained for the hydrolysis reaction, which is greatly facilitated by convenient plate-reader assays, whereas the transphosphatidylation reaction of the PLD has been much less researched. To address this void, we developed a novel high-throughput assay for the detection of the transphosphatidylation reaction in the presence of alcohol acceptors. This new assay is based on utilising amino alcohols, the natural alcohol acceptors of the bacterial PLDs, which once been exchanged for the lipid headgroup can be detected by the amine-specific reagent Fluorescamine. To achieve high-throughput capabilities, lipid substrates were coated on the bottom of plastic multi-well plates [13], avoiding detergent and organic solvents (2-phase systems), to monitor PLD rates for both hydrolysis and transphosphatidylation reactions. This novel plate reader assay was employed to evaluate the transphosphatidylation reaction of the PLD from *Streptomyces* sp., which is the most widely used PLD in biotech applications [7].

## 2. Material and Methods

### 2.1. Transphosphatidylation

For a typical assay, 10 μL of 10 mM DOPC (1,2-Dioleoyl-*sn*- glycero-3-phosphocholine) dissolved in chloroform: methanol (1:1 *v*/*v* for Costar plates or 1:1.5 *v*/*v* for BRAND plates) is added to a white 96-well plate (polystyrene). The plate is air dried on a shaker at 1200 rpm in a fumehood for a minimum of 30 min. In each well, a solution with a total volume of 50 μL containing 100 mM sodium acetate buffer at pH 5.6 (the pH employed for most synthesis reactions in organic solvents) [14,15,16], 500 mM ethanolamine (pH adjusted to 5.6) and 0.01 units of PLD from *Streptomyces* sp. (Type VII from Sigma-Aldrich, St. Louis, MO, USA) is added. The plate is shaken vigorously for 10 s before placed in a shaking incubator at 37 °C), (or 60 °C), 330 rpm for 30 min. The solutions in the wells are then removed and the wells washed three times with 100 μL of 100 mM sodium acetate buffer (pH 5.6) in order to remove any choline or ethanolamine. After three additional washes with 100 μL water, 50 μL of water is added, followed by addition of 50 μL of 5 mM Fluorescamine in acetone [17]. Fluorescamine will react with the amino moiety of 1,2-Dioleoyl-*sn*-glycero-3-phosphoethanolamine (DOPE) which has been generated by the PLD-mediated transphosphatidylation reaction with ethanolamine. The plate is shaken for 10 s before fluorescence (E_x_/E_m_ = 420/490/nm) is measured at 25 degrees over 120 min using a SpectraMax M3 plate reader (Molecular Devices), followed by an endpoint measurement after the fluorescent signal has saturated.

### 2.2. Hydrolysis

The deposition of the lipid is the same as the transphosphatidylation reaction. The hydrolysis is a two-step approach based on the detection of inorganic phosphates [18,19]. The buffer is added as a 50 μL solution containing 100 mM sodium acetate buffer at pH 5.6 and 0.02 units of PLD from *Streptomyces* sp. The plate is shaken vigorously for 10 s before incubating at 37 °C for 10 min, after which 35 µL of 1 M n-butanol is added to each well to stop further PLD hydrolysis [14]. After standing still for 5 min at room temperature, 0.3 units of acid phosphatase is added to each well, which will release phosphate from the PLD-hydrolysis product phosphatidic acid (Figure 1). The plate is again shaken on a rocker for 10 s followed by incubation at 37 °C for 30 min. The solution is transferred to a clear 96-well plate and 50 µL malachite green reagent (6 mM malachite green oxalate, 19 mM ammonium molybdate, 77 mM bismuth citrate, 2 M hydrochloric acid) is added to the plate, which will react with the released phosphate generating a change in colour [13]. The absorbance is measured at 630 nm on a spectrometer after 10 min at room temperature (23 °C); O.D. values were converted into moles using inorganic phosphate as standards [20].

### 2.3. Determination of Catalytic Rates

For the transphosphatidylation reaction, the reaction is linear up to 60 min. The reaction is stopped at 30 min, with the fluorescence signal measured converted to amount of product (DOPE) using the linear range of a calibration curve created by employing DOPE standards coated on plates. A calibration curve is generated each time a new batch of Fluorescamine solution is prepared.

The rate of hydrolysis is determined in a similar fashion, with the reaction stopped at 10 min, and the calibration curve being created using potassium phosphate as standards.

Average catalytic rates and their standard deviation (SD) are shown (some error bars may be smaller than the dot for the data points). *K*_M_ and *V*_Max_ were calculated using GraphPad Prism 9.2.0 software (GraphPad Software, San Diego, CA, USA) for fitting.

## 3. Results and Discussion

**Figure 2:**
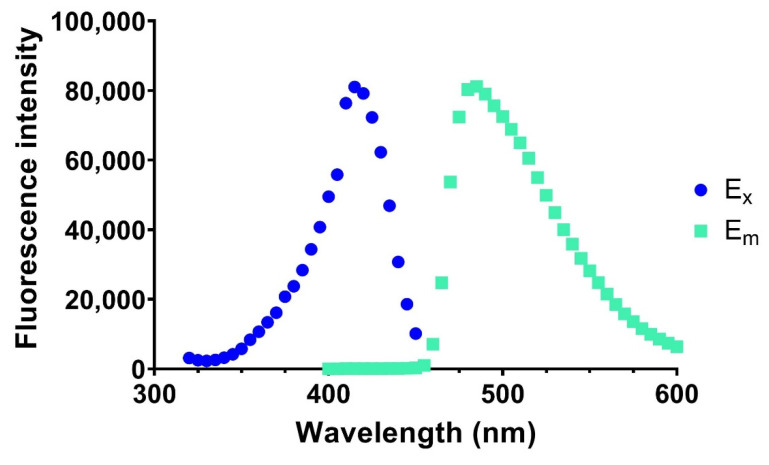
Excitation and emission spectra of Fluorescamine after reacting with PE on 96-well plates.

Fluorescamine has been employed to detect amino functionalities in high-throughput assays [21,22] as well as the distribution of phosphatidylethanolamine (PE) in cell membranes [23], but has not been employed in lipid-enzyme assays so far, with most assays employing this compound for the detection of amino functionalities in proteins and drugs [24,25]. Therefore, we investigated if this mode of detection could be applied to measure enzymatic rates in high-throughput formats such as 96-well plates. To achieve this, we modified a lipid coating method for phosphoinositides [26] by depositing DOPE in small volumes of chloroform/methanol onto the bottom of the wells of polystyrene 96-well plates. Upon drying, DOPE was reacted with a Fluorescamine solution, monitoring the excitation and emission spectra (Figure 2).

10 µL of 10 mM DOPE in 1:1 chloroform: methanol *v*/*v* were deposited on the bottom of the well and air dried on a shaker at 1200 rpm for 30 min in a fumehood. The dried wells were exposed to 50 µL of HPLC grade water and 50 µL of 5 mM Fluorescamine in acetone and reacted for 2 h at room temperature. The excitation and emission peak were determined to be between 420 nm and 490 nm, respectively.

**Figure 3:**
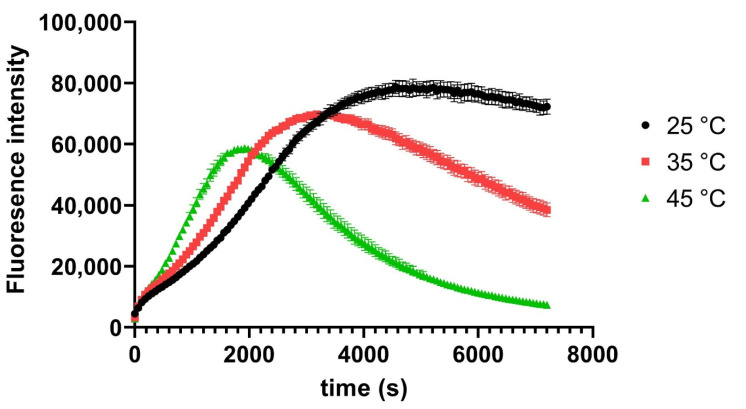
Optimisation of Fluorescamine incubation time. Data for Figure 3 are showed in the Supplementary Table S1.

The observed excitation and emission maxima were close to those reported by other investigations focussed on detecting drugs or proteins [22,27], but varied slightly indicating that the Fluorescamine coupling to a lipid has some influence on the spectroscopic parameters. We, therefore, also investigated the temperature and time dependency of the coupling reaction with Fluorescamine, which reached optimum levels after 10 min for some analytes [22,27]. As can been seen in Figure 3, the development of fluorescence over time at 25 °C reached a plateau at roughly 60 min (3600 s), with higher temperatures reaching their maximum intensity values earlier, but failing to maintain the fluorescence intensity over longer time scales. Subsequently, the Fluorescamine reaction was performed at 25 °C for all reactions.

DOPE (100 nmol) in 1:1 chloroform: methanol *v*/*v* solution was deposited on the bottom of a 96-well polystyrene plate and subsequently exposed to Fluorescamine as described in Section 2. The development of the reaction was measured over 2 h as relative fluorescence intensities (RFU) over the indicated time scale. Average RFU values from 6 experiments and their standard deviation (SD) are shown.

**Figure 4:**
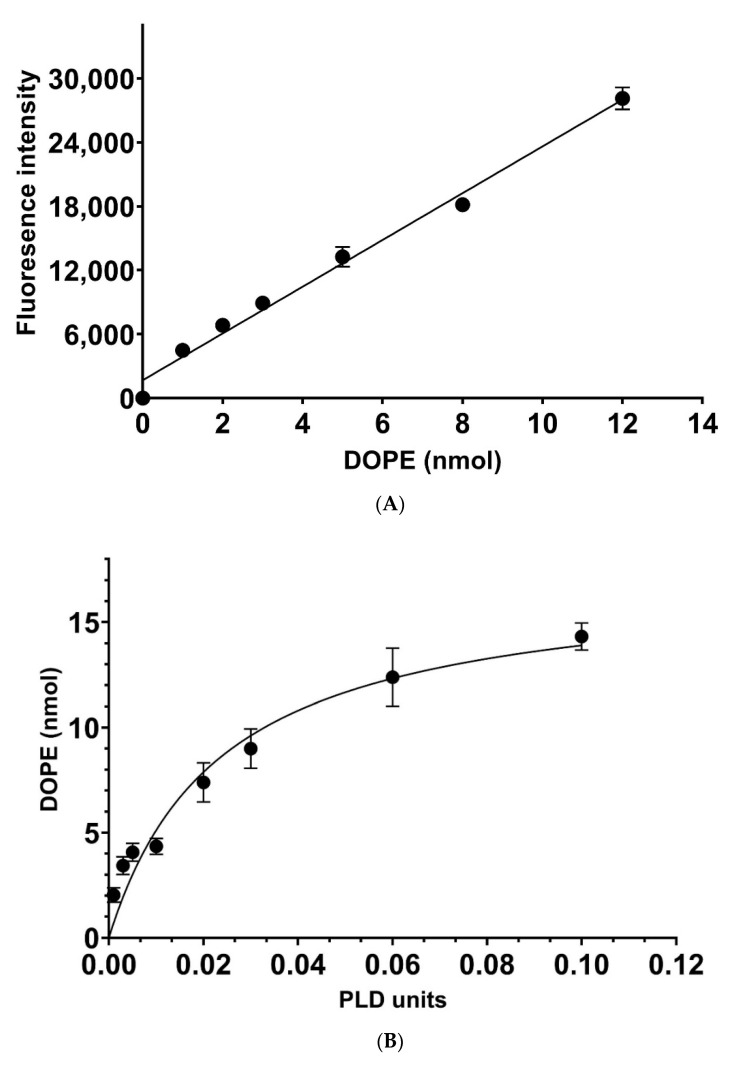

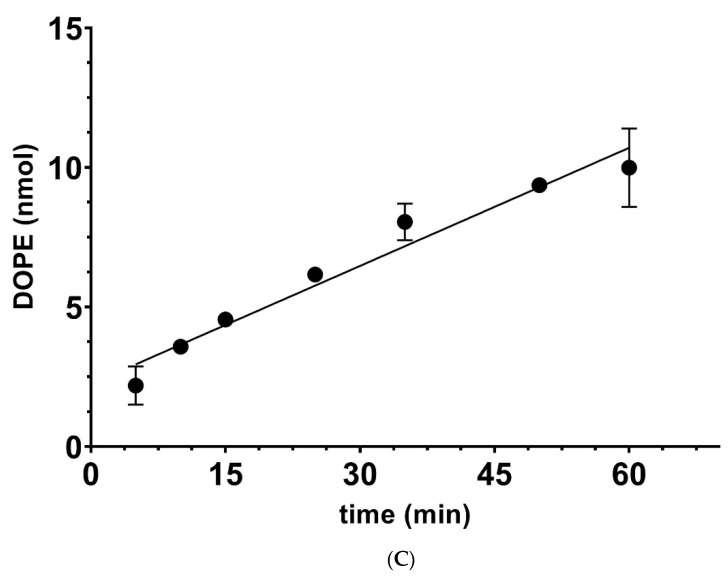
Optimisation of PLD units and reaction time. (**A**) A representative calibration curve of different amounts of coated DOPE (1, 2, 3, 5, 8, 10, 12 nmol), which were reacted with Fluorescamine as described in Section 2. Averages of 3 experiments and their SD are shown. (**B**) DOPC (100 nmol) was deposited on the bottom of a 96-well polystyrene plate and subsequently reacted with 0.5 M ethanolamine at pH 5.6 for 30 min at 37 °C in the presence of 0.001, 0.003, 0.005, 0.01, 0.02, 0.03, 0.06, and 0.1 units of PLD. The data are showing the mean of 6 experiments and the SD. (**C**) DOPC (100 nmol) was incubated with 0.5 M ethanolamine at pH 5.6 and 0.01 units of PLD at 37 °C. The data points represent incubation of 5, 10, 15, 25, 35, 50, and 60 min. Reactions were stopped and processed with Fluorescamine as described in Section 2. The amount of DOPE generated was determined from calibration curve (**A**). Average values from 6 experiments and their SD are shown. Data for Figure 4A–C are showed in the Supplementary Tables S2–S4 respectively.

Having established a detection method for coated PE in multi-well plates, we investigated its sensitivity and suitability to monitor the enzymatic rates of the transphosphatidylation reaction catalysed by the most frequently used PLD in the field (PLD *Streptomyces* sp.), which will normally employ phosphatidylcholine (PC) as a substrate and, depending on the alcohol acceptor, generate the corresponding lipid product (see Figure 1). Our data confirm that we can detect low nanomolar amounts of PE with Fluorescamine in coated polystyrene 96-well plates (Figure 4A), demonstrating a suitable detection sensitivity for the PLD-catalysed transphosphatidylation reaction with ethanolamine as the amino alcohol acceptor, which would generate PE as a product. The latter can be detected by Fluorescamine as described above, whereas the substrate PC and the released choline will be inert due to the lack of a primary amine functionality. To monitor the catalytic rates, the optimal amounts of PLD units in the transphosphatidylation reaction needed to be determined. Many PLD investigations employ high amounts of PLD to boost yields of the corresponding products in an organic solvent-based system [7,28,29]. As shown in Figure 4B, increasing units of PLD resulted in an increase in the corresponding amounts of PE, loosing linearity above 0.01 units to finally reach saturation at roughly 15% turnover. Employing 0.01 units of PLD generated products in a linear fashion over the measured time frame (Figure 4C) and was, therefore, employed in the standard assay conditions for kinetic measurements (see Section 2).

**Figure 5:**
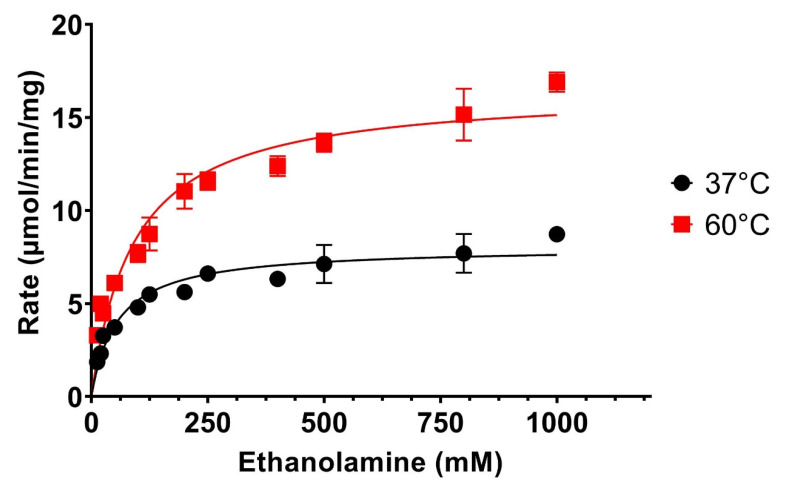
Kinetic properties of PLD *Streptomyces* sp. towards ethanolamine at 37 °C and 60 °C. Data for Figure 5 are showed in the Supplementary Table S5.

This novel application of Fluorescamine for the detection of the transphosphatidylation reactions was then employed to determine the kinetic parameters of the PLD, *K*_M_, and *V*_Max_, for the alcohol acceptor ethanolamine and the lipid substrate DOPC. The data presented in Figure 5 show the Michaelis–Menten saturation curve for the alcohol acceptor ethanolamine using DOPC as a substrate for 37 °C (black line). Non-linear fitting of the data reveals a *K*_M_ of about 45 mM and a *V*_Max_ value of about 8 µmol/min/mg at 37 °C, which is in alignment to data generated by other investigators using POPC and methanol as the alcohol acceptor [30]. These results validate this novel transphosphatidylation assay presented here and confirm that lipid coating on polystyrene multi-well plates combined with Fluorescamine detection of amino alcohol acceptors generates high-throughput kinetic data for the transphosphatidylation reaction of PLDs. This new assay makes the analysis of this so far elusive PLD reaction much more accessible, as compared to the hydrolysis reaction.

DOPC (100 nmol) was deposited in 96-well plates. A total of 0.01 units of PLD were incubated with various concentrations of ethanolamine in 100 mM pH 5.6 acetate buffer at 37 or 60 °C for 30 min. [Ethanolamine] = 12.5, 20, 25, 50, 100, 125, 200, 250, 400, 500, 800, and 1000 mM. Reactions were stopped, reacted with Fluorescamine and rates calculated as described in Section 2. Average values from 6 experiments and their standard deviation (SD) are shown.

The transphosphatidylation reaction has been employed in enzyme-assisted synthesis due to its robustness in tolerating organic solvents and higher temperatures [7,31]. We, therefore, investigated the temperature stability and changes in *K*_M_ and *V*_Max_ at 60 °C for the PLD derived from *Streptomyces* sp. As can be seen in Figure 5 (red line), *Streptomyces* sp., PLD has very good tolerance to higher temperatures, which increased its catalytic rate significantly. The corresponding *K*_M_ and *V*_Max_ values for the elevated temperature were 90 mM and 16.5 µmol/min/mg, respectively. Thus, increasing the temperature from 37 °C to 60 °C will lead to a roughly 2-fold increase of both *K*_M_ and *V*_Max_. This has important implications for the application of this enzyme in the synthesis of artificial or natural lipids, which is mostly performed in organic solvent-based systems and below 60 °C [7].

**Figure 6:**
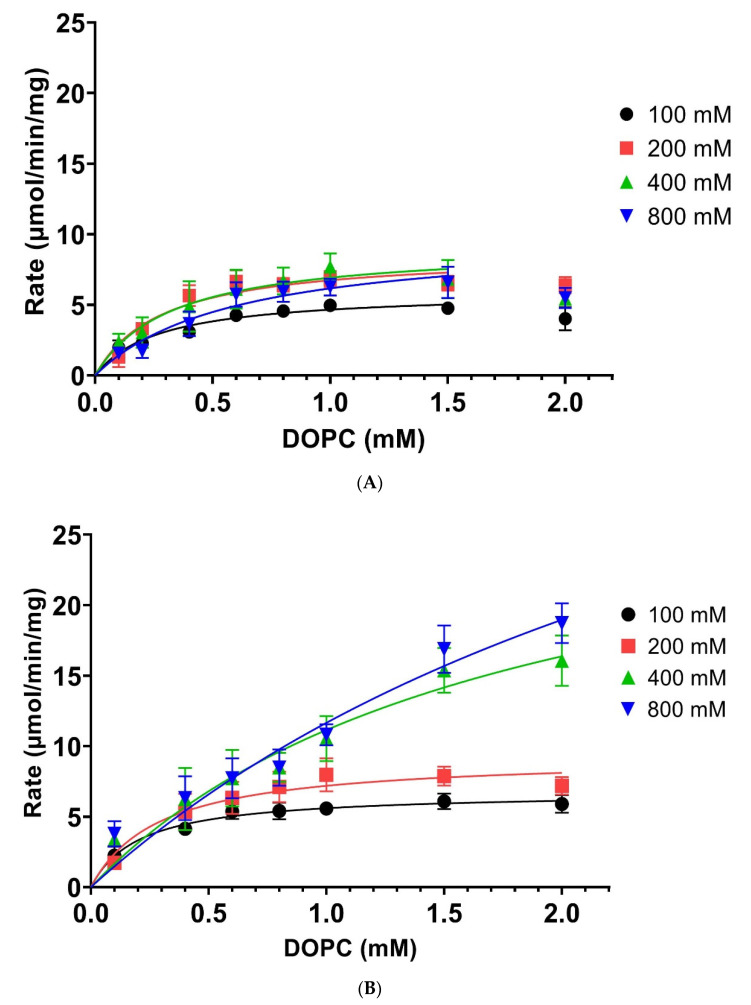
Kinetic properties of PLD *Streptomyces* sp. towards DOPC and ethanolamine at 37 °C and 60 °C. Average rates from 6 experiments and their standard deviation (SD) were fitted as described in Section 2. (**A**) Catalytic rates at 37 °C for indicated ethanolamine concentrations (100, 200, 400, or 800 mM; black, red, green, and blue colour lines) with increasing DOPC concentrations (0.1, 0.2, 0.4, 0.6, 0.8, 1.0, 1.5, 2 mM). A total of 7 data points up to 1.5 mM DOPC were fitted as described in Section 2, omitting the 2 mM DOPC concentration, which showed reduced rates. (**B**) Same conditions as in part A, except a temperature of 60 °C was employed. DOPC concentrations employed: 0.1, 0.4, 0.6, 0.8, 1.0, 1.5, and 2 mM. All data points used for fitting as described in Section 2. Data for Figure 6A,B are showed in the Supplementary Tables S6 and S7 respectively.

While it is known that higher temperatures can positively affect yields in some PLD-assisted synthesis reactions [29,32], the underlying principles governing this characteristic is not understood. The 96-well plate assay format is ideally suited to investigate the temperature effects on the kinetic properties, which is technically challenging to perform for organic solvent systems that have low boiling points (e.g., CHCL_3_, etc.). Thus, it is not surprising that *Streptomyces* sp. PLD, which is employed mostly in organic solvent systems, has not been characterised with respect to its temperature dependency. We, therefore, investigated whether the increase in catalytic rate at 60 °C is due to lipid presentation (affecting *K*_M_) or conformational changes of the PLD (affecting *V*_Max_) for both DOPC and ethanolamine (Figure 6A,B).

The PLD showed at 37 °C, a reduced rate when higher DOPC concentrations (1.5–2 mM) were applied, indicating that substrate inhibition could be possible. However, this effect is abolished at higher temperatures (60 °C), which also increases rates with higher alcohol acceptor concentrations. This characteristic could explain why enzyme-assisted synthesis applications work better at elevated temperatures and high alcohol acceptor concentrations [7]. Employing higher alcohol acceptor concentrations at 37 °C seems to be not beneficial, with *V*_Max_ values rising only slightly up to 200 mM ethanolamine concentrations, with higher ethanolamine concentrations failing to significantly increase maximum rates further (see Table 1). In contrast, the *K*_M_ values do only change significantly at 800 mM ethanolamine concentration as compared to the 200 mM concentration but are otherwise not significantly changed.

Changing the temperature to 60 °C reveals distinct effects at low and high alcohol acceptor concentrations. There is significant change observed for the *K*_M_ for alcohol acceptor concentrations higher than 400 mM (see Table 1). Whereas the *V*_Max_ values show significant changes at 100, 400, and 800 mM concentrations. If one compares the changes caused by increasing the temperature at a given alcohol acceptor concentration, it is worth noting that there is no significant difference observed for both *K*_M_ and *V*_Max_ at 200 mM concentration. All other concentrations show significant increases, but only very small ones at the lowest concentration, with much higher increases at 400 and 800 mM concentrations.

The change in both parameters as a response to elevated temperatures indicates that lipid presentation and conformational changes may occur. However, it cannot be ruled out that the high alcohol acceptor concentrations may be affecting the enzymatic properties. To investigate this further, we determined how the temperature affects the *K*_M_ for the lipid substrate in the hydrolysis reaction. Since the latter is using the same lipid substrate as the transphosphatidylation reaction, with the alcohol acceptor being replaced by water (see Figure 1), we can get an insight into the lipid presentation effect at increasing temperatures.

**Figure 7:**
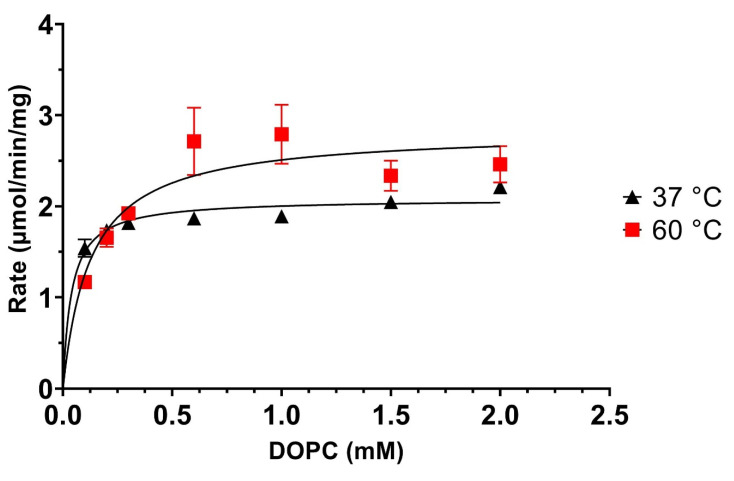
Determining the *K*_M_/*V*_Max_ for the hydrolysis reaction at 37 and 60 °C. Data for Figure 7 are showed in the Supplementary Table S8.

As can be seen from the data presented in Figure 7, the hydrolysis reaction showed only a marginal but significant increase in the rates of the reaction at higher temperatures (*V*_Max_ for DOPC being 2.08 ± 0.063 µmol/min/mg and 2.83 ± 0.21 µmol/min/mg for 37 °C and 60 °C, respectively), in line with the transphosphatidylation reaction at low alcohol acceptor concentrations. However, the curves also reveal much sharper increases of the PLD rates at low DOPC concentration when compared to the transphosphatidylation reaction (see Figure 6 for comparison). Indeed, the calculated *K*_M_ values for the lipid substrate DOPC are 0.04 ± 0.01 mM for 37 °C, whereas at 60 °C the *K*_M_ is elevated to 0.13 ± 0.04 mM, a significant increase compared to the lower temperature. This observation implies that the higher temperature reduces the binding affinity of the PLD to the DOPC substrate. Thus, it seems that the lipid affinity (*K*_M_), and not the catalytic turnover (*V*_Max_), is influenced by the increase in temperature. While there is a trend towards higher rates with elevated temperatures also visible in the hydrolysis reaction (Figure 7), compared to the transphosphatidylation reaction, this increase is much lower. This implies that the several-fold increase in catalytic activity (*V*_Max_) at higher temperatures cannot be attributed to conformational effects on the enzyme, since both hydrolysis and transphosphatidylation should have been showing similar increases. Because the *V*_Max_ increases substantially with increasing alcohol acceptor concentrations, it is likely that the PLD derived from *Streptomyces* sp. is responding to either the physical effects of the alcohols or is sensing elevated concentrations in an allosteric modulation. Given that the PLD is not only resistant, but actually very active in the presence of organic solvents, we can exclude any physical effects by the alcohols being responsible for the observed *V*_Max_ effects. Therefore, it is very likely that the *Streptomyces* sp. PLD contains another binding site for the alcohol acceptor that modulates its catalytic activity at elevated temperatures.

Different DOPC (0.1, 0.2, 0.3, 0.6, 1.0, 1.5, and 2 mM) concentrations were subjected to PLD catalysed hydrolysis as described in Section 2. Average rate values from 3 experiments and their standard deviation (SD) are shown, and subsequently fitted as described in Section 2 to obtain *K*_M_ and *V*_Max_ values.

## 4. Conclusions

We developed a novel fluorescence-based detection of the products of the PLD transphosphatidylation reaction. This method was optimised for a high-throughput plate reader assay facilitating the investigation of the effect elevated temperatures have on the kinetic properties of PLD transphosphatidylation reactions. Our assay revealed increases in *K*_M_ and *V*_Max_ at 60 °C for the PLD derived from *Streptomyces* sp. that suggest synthesis applications should be performed at elevated temperatures using suitable organic solvents. Our data also reveal that transphosphatidylation reactions will be most efficient if both substrate concentrations are high, to compensate for the increase in *K*_M_ for the lipid substrate at elevated temperatures. Comparing the transphosphatidylation and hydrolysis reactions at the different temperatures allowed us to gain an insight into the underlying principle causing the increases in lipid *K*_M_ and *V*_Max_ values at higher temperatures for the *Streptomyces* sp. PLD. Hydrolysis reactions showed increased *K*_M_ values but only marginal increases in *V*_Max_, which led to the conclusion that *K*_M_ increases observed in the transphosphatidylation reaction are caused by the elevated temperature. Since the substantial *V*_Max_ increases only occur at high alcohol acceptor concentration and at higher temperatures, it is likely that at higher temperatures, a new binding site for the alcohol acceptor is becoming accessible. Our investigation has, therefore, led to the hypothesis that the PLD derived from *Streptomyces* sp. contains an allosteric substrate binding site for its second substrate, the alcohol acceptor. While we cannot rule out the possibility that the observed allosteric site is unique for the particular PLD tested here, it is a feature affecting the most frequently employed PLD in enzyme-assisted synthesis applications and could, therefore, lead to interesting opportunities optimising the catalytic activity in recombinant and native PLDs employed for lipid synthesis applications.

## Figures and Tables

**Table 1:** Summary of kinetic parameters of PLD *Streptomyces* sp. presented in Figure 6.

[Ethanolamine] mM	100		200		400		800	
Temperature (°C)	37	60	37	60	37	60	37	60
***V*_max_ (µmol/min/mg)**	5.9 ± 0.48	6.76 ± 0.25	8.68 ± 1.0	9.29 ± 0.79	9.19 ± 0.84	30.87 ± 6.93	9.85 ± 1.49	50.88 ± 20.59
***K*_M_** **(mM)**	0.28 ± 0.07	0.21 ± 0.03	0.29 ± 0.11	0.29 ± 0.09	0.33 ± 0.09	1.77 ± 0.67	0.6 ± 0.21	3.36 ± 1.91
**Significance for *V*_max_ against 200 mM of same temperature**	S	S	NA	NA	NS	S	NS	S
**Significance for *V*_max_ against 37 °C of same concentration**	NA	S	NA	NS	NA	S	NA	S
**Significance for *K*_M_ against 200 mM of same temperature**	NS	NS	NA	NA	NS	S	S	S
**Significance for *K*_M_ against 37 °C of same concentration**	NA	S	NA	NS	NA	S	NA	S

Significance is indicated based on equal two sample *t*-test comparisons (*p* < 0.05). S = significant difference; NS = no significant difference; NA = not applicable.

## Data Availability

The data for Figure 3, Figure 4, Figure 5, Figure 6 and Figure 7 are available in the supplementary material.

## Supplementary Materials

The following supporting information can be downloaded at: https://www.mdpi.com/article/10.3390/biom12050632/s1, Table S1: Data used to plot fluorescence intensity of 100 nmol of DOPE against time at three different temperatures (Figure 3) in the article; Table S2: Data used to plot DOPE calibration curve (Figure 4A) in the article; Table S3: Data used to plot PLD units linearity testing (Figure 4B) in the article; Table S4: Data used to plot time linearity of transphosphatidylation with ethanolamine (500 mM) and 0.01 units of PLD (Figure 4C) in the article; Table S5: Data used to determine kinetic parameters of PLD *Streptomyces* sp. towards ethanolamine at 37 and 60 °C (Figure 5) in the article; Table S6: Data used to determine kinetic parameters of PLD *Streptomyces* sp. towards DOPC at 37 °C in the presence of different concentrations of ethanolamine (Figure 6A) in the article; Table S7: Data used to determine kinetic parameters of PLD *Streptomyces* sp. towards DOPC at 60 °C in the presence of different concentrations of ethanolamine (Figure 6B) in the article; Table S8: Data used to determine hydrolytic kinetic parameters of PLD *Streptomyces* sp. towards DOPC at 37 and 60 °C (Figure 7) in the article.
