# Supplementary material for: A Novel High-Throughput Assay Reveals That the Temperature Induced Increases in Transphosphatidylation of Phospholipase D Are Dependent on the Alcohol Acceptor Concentration
Source: Biomolecules. 2022 Apr 25;12(5):632. doi: 10.3390/biom12050632 (PMC9138380; doi:10.3390/biom12050632)
Supplement: Supplementary file 1 [file biomolecules-12-00632-s001.zip › biomolecules-1638883-supplementary.pdf]

Supplementary information

Table S1. Data used to plot fluorescence intensity of 100 nmol of DOPE against time at three different temperatures (**Figure 3**) in the article

Time (s)	25 (°C)		35 (°C)		45 (°C)	
	mean	SD	mean	SD	mean	SD
0	4499.194	583.8037	3274.388	32.77655	2887.817	90.33696
60	6410.611	872.7948	6899.516	737.9673	6940.78	246.4926
120	8147.934	975.2078	9044.118	1052.231	9535.673	210.9706
180	9295.609	1057.73	10566.32	1298.397	11401.24	350.092
240	10235.01	1063.371	11740.65	1302.659	12857.65	361.2206
300	11045.7	1042.004	12802.19	1221.793	14253.44	301.3538
360	11756.41	1052.38	13678.39	1245.035	15563.54	225.025
420	12626.61	954.3641	14543.25	1177.671	17183.86	342.4059
480	13272.23	892.6794	15668.27	1020.017	18832.39	140.8781
540	14053.87	861.8426	16523.84	888.1718	21046.61	394.0589
600	14803.84	892.2085	17693.75	796.0663	22784.84	382.407
660	15535.6	856.5149	18549.93	590.9706	25050.24	597.4319
720	16375.8	876.1763	19882.83	609.1348	27473.31	719.8531
780	17336.24	765.5259	21007	500.284	29885.42	867.8354
840	18142.68	803.9009	22272.25	489.8044	32060.63	991.3911
900	19076.66	777.2534	23837.3	525.7351	34318.08	1180.385
960	20014.92	773.3356	24997.46	466.9905	36393.26	1232.31
1020	20849.61	784.7835	26539.02	472.7081	38738.82	1370.207
1080	21700.13	870.6787	27839.77	591.9739	40987.14	1470.168
1140	22869.28	811.8629	29711.21	397.0424	43555.32	1670.398
1200	23901.53	908.3221	30972.95	500.2472	45399.77	1763.948
1260	25068.08	822.0312	32546.92	547.3213	47070.48	1666.302
1320	26064.3	899.9129	34249.7	716.6748	49858.46	1990.79
1380	27041.77	865.5453	35839.95	503.0979	51507.85	2009.505
1440	28495.33	886.5757	37884.42	777.1764	52998.39	1714.186

1500	29353.05	833.2467	39475.21	647.4537	54600.86	1591.408
1560	31070.28	935.4415	41490.17	812.0231	55691.64	1286.192
1620	32024.43	987.9983	42895.8	920.8245	57149.53	963.1732
1680	33494.65	993.1041	45110.91	926.9754	57186.26	874.9758
1740	34785.95	1102.562	46527.35	981.5254	58111.29	552.0055
1800	36230.77	995.759	48087.88	1125.188	58412.36	479.0628
1860	37629.19	927.0489	50607.32	1071.173	58357.83	229.5984
1920	39085.76	855.6082	52395.27	1100.189	58721	122.0745
1980	40576.44	844.5278	54367.62	1106.565	58460.65	250.8308
2040	42072.1	1152.673	56339.64	856.0285	58100.84	403.6967
2100	43672.61	1033.899	57542.89	705.5685	57584.07	665.0955
2160	45072.7	1162.284	59117.06	677.0851	56555.11	668.3813
2220	46188.52	1070.483	60229.16	617.3986	56264.04	909.369
2280	48102.72	1082.501	61298.11	598.6229	55538	1369.406
2340	49509.96	1181.327	62798.64	660.3454	55027.11	1410.801
2400	51610.06	900.5501	63644.64	466.9333	53988.83	1592.302
2460	52620.3	1107.232	64458.24	416.5623	53160.03	1627.946
2520	54347.8	1117.643	64686.77	380.9819	52058.36	1853.41
2580	55780.8	1228.864	65795.17	419.9381	51127.54	1903.031
2640	57334.06	1387.676	66099.79	361.1667	50177.13	1976.025
2700	58288.67	1452.933	67289.8	644.3166	49165.33	1907.718
2760	60137.74	1228.564	67548.01	480.2527	48167.55	2140.451
2820	61617.54	1408.637	68268.24	172.57	46987.44	2108.288
2880	62577.76	1504.03	68623.22	262.7061	45717.01	2108.823
2940	63711.73	1320.712	68414.29	532.8977	44654.28	2231.181
3000	64678.92	1590.048	69088.4	399.0363	43584.45	2321.327
3060	65608.18	1392.6	69479.55	409.7262	42635.8	2452.376
3120	66495.69	1559.772	69437.23	680.8645	41536.89	2282.98
3180	67267.02	1303.772	69821.77	677.3014	40240.43	2311.242
3240	68194.04	1935.837	69479.69	837.0908	39349.83	2426.374

3300	69036.93	1546.351	69300.05	916.6825	38132.51	2430.494
3360	70102.72	1592.959	69808.18	880.9682	37069.1	2434.517
3420	70981.84	1942.372	69609.96	1088.704	36207.63	2304.263
3480	71141.51	1555.671	69283.44	1066.315	34909.75	2426.253
3540	71828.66	1648.772	68696.39	1163.562	34134.35	2370.789
3600	72835.61	1558.228	68617.04	1264.179	33402.35	2259.382
3660	73337.6	1689.069	68325.75	1383.779	32392.22	2224.29
3720	73658.95	1862.39	68347.86	1326.547	31553.16	2331.793
3780	74316.22	1804.641	68102.75	1469.793	30533.99	2155.366
3840	74669.05	1999.504	67513.21	1417.39	29581.99	2289.17
3900	75468.61	1802.4	67338.72	1698.281	28808.81	2090.136
3960	75360.3	2085.813	66495.91	1796.579	28235.87	2055.45
4020	75899.67	2018.168	66536.75	1789.366	27374.11	2101.05
4080	76216.6	2171.51	66447.64	1856.362	26692.47	2156.67
4140	76576.45	2038.529	65003.75	1726.372	26038.6	2000.17
4200	76700.9	1896.615	64755.99	1594.223	25179.33	1959.182
4260	77298.56	1880.895	64384.7	1813.054	24302.56	1909.258
4320	76913.39	2223.14	64083.1	2022.845	23863.43	1808.231
4380	77376.64	2408.338	63664.17	2071.28	23291.63	1793.733
4440	77700.33	2153.691	63422.92	1955.331	22447.06	1680.828
4500	77859.61	2176.443	63126.15	1865.832	21748.91	1553.879
4560	78733.58	2155.231	61889.98	2092.759	21337.65	1589.524
4620	78165.58	2536.668	61453.13	2005.026	20687.25	1604.772
4680	78200.29	2639.106	60468.62	2162.184	19984.79	1430.534
4740	78245.53	2389.328	60624.35	2186.284	19630.03	1454.946
4800	77666.43	2367.846	59837.15	2081.61	19037.83	1309.198
4860	78452.33	2832.514	59298.39	2023.749	18611.16	1410.634
4920	78129.01	2291.157	58722.61	2232.001	17925.29	1189.729
4980	77875.59	1904.885	58513.69	2180.713	17504.77	1314.267
5040	78268.01	2302.4	57856.06	2255.51	17010.22	1308.697

5100	77733.09	2552.368	57336.15	2345.419	16559.59	1096.287
5160	78285.51	2426.077	56757.54	2378.633	16046.31	1054.169
5220	78565.55	2311.877	56061.36	2183.949	15763.91	1072.56
5280	77454.4	2601.368	55616.74	2336.459	15348.56	1046.005
5340	78074.91	2703.035	54730.62	2287.293	14848.53	892.086
5400	77879.06	2433.179	54207.28	2276.007	14523.37	899.842
5460	77894.07	2432.536	54012.76	2350.431	14082.05	724.5971
5520	77662.16	2746.508	53084.93	2491.811	13830.09	816.5219
5580	76663.96	2757.643	52299.13	2448.305	13557.01	794.8246
5640	77656.48	2697.215	51974.09	2579.856	13125.75	807.8704
5700	76502.67	2941.251	51386.76	2155.688	12819.65	637.6701
5760	76916.97	2629.187	51117.23	2249.964	12436.24	669.0284
5820	76936.12	2841.956	50151.4	2342.688	12246.92	519.5943
5880	77113.92	2787.696	49916.14	2431.344	11967.68	553.8175
5940	76437.76	2668.789	49090.84	2424.41	11647.3	489.0663
6000	76506.38	2363.458	48455.91	2290.459	11380.58	382.1291
6060	76137.16	2635.957	48648.22	2466.849	11133.05	454.139
6120	75528.54	2369.322	47671.07	2420.811	10850.89	468.4409
6180	75411.69	2482.373	47005.13	2341.687	10592.48	344.8467
6240	75663.72	2616.013	46689.59	2436.367	10509.21	391.4709
6300	75171.59	2306.776	46007.43	2316.634	10174.59	301.9067
6360	75363.33	2563.506	45542.7	2524.974	9945.051	263.014
6420	74714.64	2600.661	44695.29	2270.446	9767.512	285.6759
6480	74471.85	2673.482	44040.54	2345.156	9553.648	282.0914
6540	74535.13	2321.205	44172.21	2173.539	9285.669	302.7513
6600	74417.56	2565.957	43192.4	2188.527	9137.308	267.5367
6660	73603.32	2373.818	42647.32	2238.829	8974.514	244.2455
6720	73985.85	2522.79	42148.46	2250.868	8730.317	148.9735
6780	73310.16	2237.392	41825.85	2339.482	8570.141	286.2375
6840	73298.09	2053.997	41170.78	2233.298	8383.487	290.4452

6900	73085.39	2426.733	40587.31	2206.575	8283.886	225.023
6960	72787.03	1997.688	39967.03	2241.098	8000.846	254.401
7020	72486.91	2107.95	39713.11	2190.575	7880.93	248.6714
7080	72074.67	2564.624	39387.34	2254.944	7677.021	280.7476
7140	71949.4	2186.319	38979.23	2209.668	7628.111	289.7193
7200	72272.39	2438.546	38476.55	2268.112	7380.346	181.6592

Table S2. Data used to plot DOPE calibration curve (**Figure 4A**) in the article

DOPE (nmol)	Fluorescence intensity	
	mean	SD
0	0	0
1	4477.29	504.7434
2	6835.147	175.2168
3	8918.576	483.774
5	13261.22	927.135
8	18153.63	245.983
12	28126.96	1038.017

Table S3. Data used to plot PLD units linearity testing (**Figure 4B**) in the article

PLD units	DOPE (nmol)	
	mean	SD
0.001	2.031274	0.341593
0.003	3.432972	0.422641
0.005	4.065907	0.42714
0.01	4.346704	0.376864
0.02	7.386858	0.932969
0.03	8.99207	0.939238
0.06	12.38254	1.377784
0.1	14.31833	0.646128

Table S4. Data used to plot time linearity of transphosphatidylation with ethanolamine (500 mM) and 0.01 units of PLD (**Figure 4C**) in the article

Time (min)	DOPE (nmol)	
	mean	SD
5	2.182443	0.684717
10	3.578789	0.216799
15	4.550812	0.261053
25	6.165255	0.25246
35	8.049491	0.659589
50	9.365412	0.195342
60	9.992835	1.40579

Table S5. Data used to determine kinetic parameters of PLD *Streptomyces* sp. towards ethanolamine at 37 and 60 °C (**Figure 5**) in the article

Ethanolamine (mM)	37 (°C)		60 (°C)	
	mean	SD	mean	SD
12.5	1.858035	0.13139	3.295453	0.24411
20	2.321125	0.095066	4.988083	0.371909
25	3.271484	0.05609	4.479388	0.298757
50	3.718456	0.280418	6.107876	0.227058
100	4.795751	0.251637	7.690892	0.466664
125	5.48729	0.136978	8.733923	0.888151
200	5.621171	0.263639	11.02671	0.935164
250	6.610619	0.184781	11.58572	0.475737
400	6.319729	0.146967	12.38867	0.532889
500	7.12515	1.022074	13.64659	0.489998
800	7.695747	1.036036	15.15168	1.392346
1000	8.724178	0.274728	16.89148	0.523712

Table S6. Data used to determine kinetic parameters of PLD *Streptomyces* sp. towards DOPC at 37 °C in the presence of different concentrations of ethanolamine (**Figure 6A**) in the article

	Ethanolamine (mM)							
	100		200		400		800	
DOPC (mM)	mean	SD	mean	SD	mean	SD	mean	SD
0.1	1.975285	0.502566	1.314223	0.718753	2.36417	0.581278	1.561022	0.333776
0.2	2.279034	0.163405	3.299822	0.332536	3.031593	1.08272	1.711545	0.482574
0.4	3.076919	0.331298	5.651131	0.737525	4.879844	1.789135	3.658223	0.875492
0.6	4.256201	0.310297	6.656425	0.793591	6.106299	1.369577	5.730639	0.869276
0.8	4.559929	0.214148	6.418271	0.479008	6.689419	0.95179	5.92976	0.719898
1.0	4.964193	0.206302	6.827214	0.562191	7.690704	0.953211	6.25422	0.593052
1.5	4.763865	0.43034	6.534767	0.525872	6.818682	1.352979	6.584361	1.111261
2.0	4.014971	0.829897	6.40121	0.570206	5.415506	0.398558	5.484435	0.712584

Table S7. Data used to determine kinetic parameters of PLD *Streptomyces* sp. towards DOPC at 60 °C in the presence of different concentrations of ethanolamine (**Figure 6B**) in the article

	Ethanolamine (mM)							
	100		200		400		800	
DOPC (mM)	mean	SD	mean	SD	mean	SD	mean	SD
0.1	2.2283	0.646326	1.72445	0.35687	3.379033	0.388723	3.787662	0.889216
0.4	4.134581	0.477251	5.336768	1.285188	6.251936	2.202897	6.310172	1.556995
0.6	5.36889	0.529432	6.332653	1.134589	7.726542	1.998625	7.723566	1.420609
0.8	5.403917	0.593516	7.107206	1.079323	8.494565	1.025114	8.480869	1.288735
1.0	5.580245	0.41319	7.97298	1.178932	10.53543	1.596381	10.80535	0.747081
1.5	6.086528	0.54639	7.879444	0.679168	15.38723	1.582769	16.88363	1.676352
2.0	5.902959	0.62304	7.190198	0.622239	16.06952	1.787034	18.73032	1.408083

Table S8. Data used to determine hydrolytic kinetic parameters of PLD *Streptomyces* sp. towards DOPC at 37 and 60 °C (**Figure 7**) in the article

DOPC (mM)	37 (°C)		60 (°C)	
	mean	SD	mean	SD
0.1	1.538938	0.096637	1.166421	0.065813
0.2	1.733576	0.013009	1.653721	0.101554
0.3	1.81498	0.055326	1.922722	0.067046
0.6	1.866105	0.015675	2.71226	0.369094
1.0	1.888357	0.020374	2.790143	0.323195
1.5	2.045673	0.014832	2.334955	0.166705
2.0	2.210454	0.013654	2.460723	0.200037
